# Solid-Liquid Phase Diagram of the Dimethyl + Dipropyl Adipates System: Application to Low-Temperature Thermal Energy Storage

**DOI:** 10.1007/s10765-025-03598-2

**Published:** 2025-07-10

**Authors:** Maria C. M. Sequeira, Timur Nikitin, Fernando J. P. Caetano, Hermínio P. Diogo, João M. N. A. Fareleira, Rui Fausto

**Affiliations:** 1 Centro de Química Estrutural, Institute of Molecular Sciences, Departamento de Engenharia Química, Instituto Superior Técnico, Universidade de Lisboa, Av. Rovisco Pais, 1049-001 Lisbon, Portugal; 2 CQC-IMS, Departamento de Química, Universidade de Coimbra, 3004-535 Coimbra, Portugal; 3 Departamento de Ciências e Tecnologia, Universidade Aberta, 1269-001 Lisbon, Portugal; 4 Centro de Química Estrutural, Institute of Molecular Sciences, Instituto Superior Técnico, Universidade de Lisboa, Av. Rovisco Pais, 1049-001 Lisbon, Portugal; 5 ERA-Chair Spectroscopy@IKU, Faculty of Sciences and Letters, Department of Physics, Istanbul Kultur University, Ataköy Campus, 34156 Bakirköy, Istanbul Turkey

**Keywords:** Adipates, Solid–liquid phase diagram, Phase Change Material (PCM), Eutectic, Low-temperature energy storage (TES)

## Abstract

The present study is the continuation of our research work on di-*n*-alkyl adipates and their potential as phase change materials (PCM) for low-temperature thermal energy storage (TES). The solid–liquid phase diagram for the binary system composed of dimethyl adipate (DMA) and dipropyl adipate (DPA) is presented and analysed. In a previous study, we explored a particular binary system of *n*-alkyl adipates, namely diethyl and dibutyl adipates, and demonstrated that these compounds possess underappreciated potential as PCMs at sub-zero temperatures. The goal of the current work is to expand on this research by contributing new phase equilibrium data and deepening our understanding of the fundamental thermodynamics governing low-temperature phase behaviour in di-*n*-alkyl-adipates. The phase diagram for the DMA + DPA binary system was obtained using three complementary techniques: differential scanning calorimetry (DSC), hot-stage microscopy (HSM), and Raman spectroscopy. DSC analysis of sixteen compositions, including the two pure compounds, provided both the temperature and enthalpy values for the solid–liquid and solid–solid phase transitions. The binary system displays eutectic behaviour at low temperatures, with the eutectic point found at 252.83 K and a composition of approximately x_DPA_ = 0.77. Raman spectroscopy confirmed that the system is characterized by a non-isomorphic eutectic phase diagram, indicating differences in the crystal structures of the solid phases. The *liquidus* line of the binary phase diagram was successfully described using a suitable fitting equation, yielding a root mean square deviation of 0.65 K, indicating excellent agreement between the experimental data and the theoretical model. This fitting also allowed an accurate prediction of the eutectic composition and temperature. A Tammann diagram is also presented, further confirming the eutectic composition and associated enthalpy. This work addresses a gap in the literature by presenting, for the first time, the solid–liquid phase equilibrium behaviour of the DMA + DPA binary system (including the detailed solid–liquid phase diagram of the system). The findings provide valuable insight into the potential use of this system as PCM for sub-zero TES applications, supporting their consideration in future thermal energy storage technologies.

**Supplementary Information:**

The online version contains supplementary material available at 10.1007/s10765-025-03598-2.

## Introduction

As part of an ongoing program developed by our group to identify suitable phase change materials (PCM) for applications at sub-zero temperatures, we have investigated a range of systems, including a binary mixture of di-*n*-alkyl adipates [1] and several *n*-alkane mixtures [2–4].

The interest in *n*-alkyl adipates is illustrated by our previous studies on their thermophysical properties [5–8], which have demonstrated promising potential for practical deployment in thermal energy storage (TES) systems. These compounds exhibit a combination of favourable characteristics for TES applications, including low toxicity, high chemical and thermal stability, cost-effectiveness, and environmental compatibility, making them strong candidates for use in industrial TES applications [8, 9].

Consequently, those compounds became an interesting subject for solid–liquid phase equilibrium studies, which are fundamental for assessing their actual potential to be PCM for low-temperature TES applications. Our initial work examined the diethyl–dibutyl adipate binary system, which was found to be a non-isomorphous eutectic system with an adequate eutectic temperature for low-temperature applications [1]. Additionally, the enthalpy of fusion found for the eutectic composition indicated a good energy storage capacity, which motivated the extension of this research. The present study is the continuation of that work on *n*-alkyl adipates, in this case focussing on the binary mixture of dimethyl adipate (DMA) and dipropyl adipate (DPA).

In the course of our previous studies on solid–liquid phase equilibria in *n*-alkanes, one particularly interesting result was the confirmation of earlier findings by other authors [10, 11], which suggested that the parity of the carbon chain length in linear alkanes—that is, whether the number of carbon atoms is even or odd—plays a crucial role in the solid–liquid phase behaviour of their binary mixtures. In fact, the different characteristics in the number of carbon atoms of the studied *n*-alkane mixtures yielded eutectic systems, congruent melting solid solutions, and peritectic phase diagrams [2–4], thereby reinforcing the structural correlation between chain parity and phase behaviour.

At this stage, it is interesting to note that the first di-*n*-alkyl adipates system studied, namely, diethyl + dibutyl adipates [1], features components whose alkyl groups contain even number of carbon atoms in their chains. Interestingly, this system was found to exhibit a well-defined non-isomorphic eutectic phase diagram [1], a behaviour that closely mirrors that observed in previously studied binary mixtures of *n*-alkanes with even–even carbon chains. Thus, one of the main motivations for the present work was to further explore the potential relationships between molecular characteristics of these adipate systems (particularly alkyl chain parity) and the type of solid–liquid phase diagram they exhibit.

**Fig. 1:**
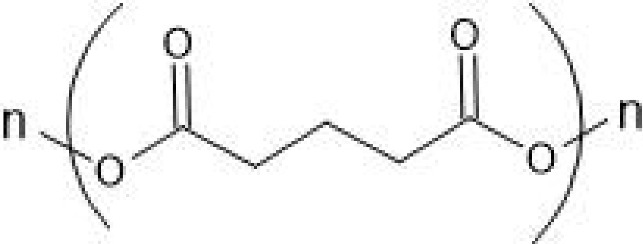
Generic molecular structure of *n*-alkyl adipates

Considering the structure of di-*n*-alkyl adipates, their only differentiating characteristic arises from the alkyl terminal substituent groups. Nevertheless, the adipate central part of these molecules (as illustrated in Fig. 1) likely plays a significant role in governing the intermolecular forces within these mixtures. The stereochemical relationship between the alkyl substituents and the central adipate part of the molecule appears to influence the physical properties of the pure compounds, particularly those related to solid–liquid phase behaviour. For example, dimethyl adipate shows the highest melting temperature around 281 K, while the other adipates (diethyl, dipropyl, and dibutyl) have sub-zero melting points around 250 K. Interestingly, dimethyl and dibutyl adipates appear to exhibit polymorphic forms, whereas diethyl and dipropyl adipates did not evidence such behaviour under the experimental conditions employed in both our previously published work [1], and in the present study.

These observations suggest that dimethyl adipate, with the smallest alkyl chain, exhibits stronger intermolecular forces, as reflected by its higher melting point. In contrast, dibutyl adipate, which has the longest alkyl chain among the four adipates studied, exhibits a significantly lower melting point, indicating weaker intermolecular interactions relative to those in dimethyl adipate. Yet intriguingly, dibutyl adipate also displays two polymorphic forms. This behaviour may stem from the increased conformational flexibility of its longer alkyl chain, which affords the molecule greater freedom to adopt alternative packing arrangements in the solid state. In contrast, diethyl and dipropyl adipates do not exhibit any polymorphic transitions under the experimental conditions explored. Considering the melting point as an indicator of the interactions between the adipate central part and the alkyl substituents, dimethyl adipate stands out due to its unusually higher melting temperature, suggesting strong cohesive interactions within the crystal. For the remaining three adipates, diethyl, dipropyl, and dibutyl, the interactions strength appears to be more comparable, possibly reflecting a balance between alkyl chain length and molecular packing efficiency.

Given the potential relevance of predicting the phase equilibrium characteristics based on the odd–even number of carbon atoms of the substituents in *n*-alkyl adipates, the present study continues previous efforts to elucidate the relationship between molecular structure and the solid–liquid phase diagrams. While earlier work focussed primarily on *n*-alkane mixtures, the current work extends this framework to a consistent group of binary mixtures of di-*n*-alkyl adipates, thereby broadening the scope and applicability of these correlations for the development of potential new PCM.

Furthermore, in line with our overarching goal of identifying suitable materials for thermal energy storage applications at sub-zero temperatures, the construction of an accurate solid–liquid binary phase diagram is essential. Such diagrams provide critical insights into the interactions between the components of the mixture, miscibility, and the phase change behaviour, which are key parameters for optimizing TES systems and enhancing the performance of the PCM. Thus, in this work we present the solid–liquid phase diagram for the binary system composed of dimethyl and dipropyl adipates. The construction of the diagram was carried out using a combined analytical approach employing Differential Scanning Calorimetry (DSC), Hot-Stage microscopy (HSM), and Raman spectroscopy. This integrative approach ensures an accurate construction of the solid–liquid phase equilibrium diagram, offering valuable insights for low-temperature TES applications. Additionally, we examined also another fundamental property for PCM applications: the enthalpy of the solid–liquid phase transitions, which directly influences the energy storage capacity of the material.

Building upon our earlier findings for the binary system of diethyl and dibutyl adipates (both with even-numbered alkyl substituents), we now extend our investigation to a system featuring odd-numbered alkyl chains in both components. This enables a more comprehensive understanding of how alkyl chain parity influences the phase behaviour of di-*n*-alkyl adipates and their viability as functional PCMs.

## Experimental

### Materials

The samples used in this work were acquired from Aldrich and TCI Chemicals, with a nominal purity characterized by a mass fraction of 99.9 % (DMA) and 99 % (DPA). Due to its relatively high moisture content, DMA was dried using 4 nm molecular sieves prior to use and was not subjected to any further treatment. DPA, on the other hand, was used as received, based on the high purity specified by the supplier and its low water content, which was verified immediately prior to the experimental measurements. The water content of both compounds was determined using a Karl-Fischer 831 KF Coulometer from Metrohm.

To achieve the highest accuracy in composition, the binary mixtures for this investigation were prepared gravimetrically using a Mettler Toledo MS205DU micro balance with a precision of ± 0.01 mg. The key characteristics of the materials used (DMA; DPA) are summarized in Table 1.

**Table 1:** Characterization of the liquids used in this work

Name	CAS number	Supplier	Lot Number	Water content (mg·kg^−1^)^a^	Purity (mass fraction)^b^ (%)
dimethyl adipate (DMA)	627-93-0	Aldrich	10811BJ499	76.4	99.9
dipropyl adipate (DPA)	106-19-4	TCI Chemicals	GF01	51.9	99

^a^Water content as measured in situ; ^b^Purity as stated in the corresponding analysis certificate.

### Techniques

#### Differential Scanning Calorimetry (DSC)

The calorimetric analyses were conducted using a Differential Scanning Calorimeter (DSC), model 2920 MDSC from TA Instruments Inc. A comprehensive description of the experimental procedure is available in references [1–4], but a concise overview is provided here. Sample masses ranging from 4.0 to 10.0 mg were placed in aluminium pans, sealed in air, and weighed with an accuracy of ± 1.0 × 10^–4^ mg using a Mettler UMT2 ultra-micro balance. These samples were then analysed using DSC at a scanning rate of *β* = 5 K·min^–1^. Helium gas (Air Liquide N55) was used as a purge, at a flow rate of 30 cm^3^·min^−1^. To correct the baseline, a scan was performed using an empty pan across the same temperature range. The temperature and heat flow scales of the instrument were calibrated at various heating rates, based on the onset of the fusion peaks of high-purity standards. Further details regarding the calibration procedure may be found in reference [12].

#### Hot-Stage Microscopy (HSM)

Polarized optical microscopy was carried out using an Olympus BX51 optical microscope. Temperature adjustments and stabilization were controlled by a Linkam LTS360 cryostage, cooled with liquid nitrogen, and monitored with a platinum resistance thermometer. The liquid samples were initially placed on a glass plate, covered with a second glass, and cooled at a rate of 15 K·min^−1^. Once the samples solidified, their microstructure was observed and captured using an Olympus C5060 wide zoom camera for both images and/or videos. The images were taken at a magnification of 250×, covering the temperature range from 173.15 K to 293.15 K, with a heating rate of 15 K·min^−1^.

#### Raman Spectroscopy

Raman spectra were recorded using a Horiba LabRam HR Evolution micro-Raman system, which employed a solid-state laser (*λ* = 532 nm, ~ 5 mW power on the sample) for excitation. A 50× objective lens was used to focus the laser on the sample, with a laser spot diameter of approximately 1 μm. Calibration of the system was performed using a silicon crystal, with the reference band observed at 520.5 cm^–1^. The spectra presented here were typically acquired with an integration time of 5 s and averaged over 10 accumulations. The spectral resolution was approximately 5 cm^–1^.

Temperature-dependent measurements were conducted with an accuracy of about 0.01 K, using a Linkam Scientific Instruments setup, which included a THMS 600 stage, an LNP95 cooling system, and a T95-PE Linkpad control unit. To investigate the thermal behaviour and phase transitions of the samples, the mixtures were cooled to complete solidification at 193.15 K, with an average cooling rate of ca. 10 K·min^−1^. Subsequently, the samples were heated at 5 K·min^−1^ (to align with DSC results) until melting, and Raman spectra were recorded at various temperatures. Spectra were also collected at room temperature prior to the cooling of the samples.

## Results and Discussion

Designing a binary phase equilibrium diagram for a system is a complex process. These diagrams provide crucial information about phase transitions, including solid–solid (S–S) and solid–liquid (S–L) transformations, as well as the coexistence of different phases in equilibrium. Though DSC appears as one of the most powerful techniques receiving application in the construction of phase diagrams, additional complementary techniques are essential to accurately characterize the system’s phase behaviour.

At low temperatures, experimental challenges become usually more pronounced, and conventional techniques such as X-ray analysis may not be feasible or practical. To address these challenges, our research group [1–3] has successfully integrated HSM and Raman spectroscopy, as complementary experimental techniques to supplement DSC data. This approach enables the construction of solid–liquid phase diagrams for binary systems, facilitating the evaluation of their potential as PCM for TES at low temperatures.

### Differential Scanning Calorimetry (DSC)

**Fig. 2:**
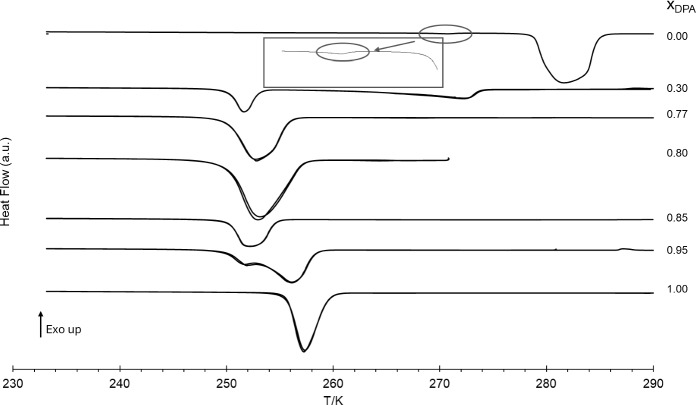
DSC heating curves of some selected binary mixtures, with compositions indicated by the x_DPA_ molar fraction. The scanning rate was *β* = 5 K·min^–1^ (exo up). Two consecutive heating runs are presented for each composition

DSC heating curves for the pure compounds, DMA and DPA, and for three of their binary mixtures are shown in Fig. 2. The remaining DSC heating curves for the studied binary mixtures are presented in section S1 of the Supplementary Information.

The experimental onset temperature, *T*_*onset*_, maximum temperature, *T*_*max*_, and enthalpy of fusion values, *Δ*_*fus*_*H,* are presented in Table 2 for the most relevant samples. These values were obtained using the average values of two consecutive cycles. The complete data for all the studied mixtures are presented in Table S1 of section S1 in the Supplementary Information.

**Table 2:** DSC data for pure dimethyl adipate (DMA), pure dipropyl adipate (DPA), and three of their binary mixtures, including the onset temperatures, *T*_*onset*_, maximum peak temperatures, *T*_*max*_, and the corresponding enthalpies of fusion, Δ_fus_*H*, at atmospheric pressure, 0.1 MPa

*X*_DPA_	DSC 1st peak					DSC 2nd peak		
	*T*_onset_/K	*T*_max_/K			Δ_fus_*H*/J·g^–1^	*T*_onset_/K	*T*_max_/K	Δ_fus_*H*/J·g^–1^
0	268.49		270.35	0.9		278.62	281.08	167.9
0.300	250.14		251.91	55.2		265.67	272.65	64.2
0.773	250.22		252.83	130.8		–	–	–
0.800	250.21		253.00	126.8		–	–	–
0.836^b^	250.33		252.19	127.3		–	–	–
0.948	249.91		251.8	–		252.2	256.11	129.3^a^
1	255.71		257.29	124.0		–	–	–

^a^Enthalpy value for the overlapped peaks; ^b^The DSC thermogram indicates that this composition corresponds to the *liquidus* line as attributed in the solid–liquid phase diagram (Fig. 13); Expanded uncertainties for a 95 % confidence level (*k* = 2): *U*(*x*) = 0.00016; *U*(*T*) = 0.16 K; *U*(Δ_fus_*H)* = 3.4 J·g^–1^ (see Supporting Information – S4)

The scanning rate was *β* = 5 K·min^–1^

Figure 2 presents DSC thermograms for samples with different molar fractions of dipropyl adipate (DPA), x_DPA_. The DSC thermograms for the binary mixtures exhibit a sharp and consistent endothermic peak at approximately 252 K, corresponding to the eutectic transition. This peak remains invariant across different compositions, underscoring the characteristic behaviour of a non-isomorphic eutectic system. Additionally, a second endothermic peak is observed, associated with the melting of the component present in excess. This peak appears at a temperature lower than that of the corresponding pure component. As the mole fraction of the component with higher concentration increases, approaching the value of 1, this second peak shifts progressively to the value of the pure component, reflecting the changing composition of the mixture. The mixture with x_DPA_ = 0.77, apparently corresponding to the eutectic composition, presents sharp endothermic peak, with the lowest melting temperature among the other studied mixtures and a relatively high enthalpy of fusion, both of which are consistent with the behaviour expected at the eutectic point. In contrast, the mixture with molar fraction x_DPA_ = 0.84 also show a single endothermic peak, but it is broader, occurs at a higher melting temperature, and is associated with a lower enthalpy of fusion, indicating a deviation from the eutectic composition.

The pure compounds also exhibit different thermal behaviours: for dimethyl adipate (DMA) two peaks were detected, whereas for DPA only one peak was observed. The first peak in the DMA thermogram (see zoomed region in Fig. 2) is barely detectable, as reflected by its very low enthalpy value (Table 2), indicating a very subtle phase transition. This behaviour is further analysed in the context of the results obtained from the other two techniques used in this study (HSM and Raman Spectroscopy), which are discussed in Sects. 3.2 and 3.3, respectively.

Further analysis of the thermal behaviour of the studied binary mixtures will be considered in the next sections, where HSM and Raman spectroscopy results are used to complement the DSC data. The binary phase diagram presented in Sect. 3.4 is the outcome of the comprehensive analysis of the studied system combining insights from all three different techniques used.

### Hot-Stage Microscopy (HSM)

Hot-Stage Microscopy is especially valuable for visual identification of S–S phase transitions (including polymorphism), as well as S–L transitions. In fact, polarized optical microscopy enables a clearer observation of events involving changes in crystalline structure by enhancing contrast and revealing distinct birefringence patterns. This visual identification is very helpful in assigning specific phase transitions to the different DSC peaks and, consequently, to identify the different regions within the binary phase diagram.

The obtained HSM images for the pure compounds, DMA and DPA, and for their most relevant binary mixtures are presented in Figs. 4,5,6, and 7. The remaining HSM images are presented in section S2 of the Supplementary Information.

**Fig. 3:**
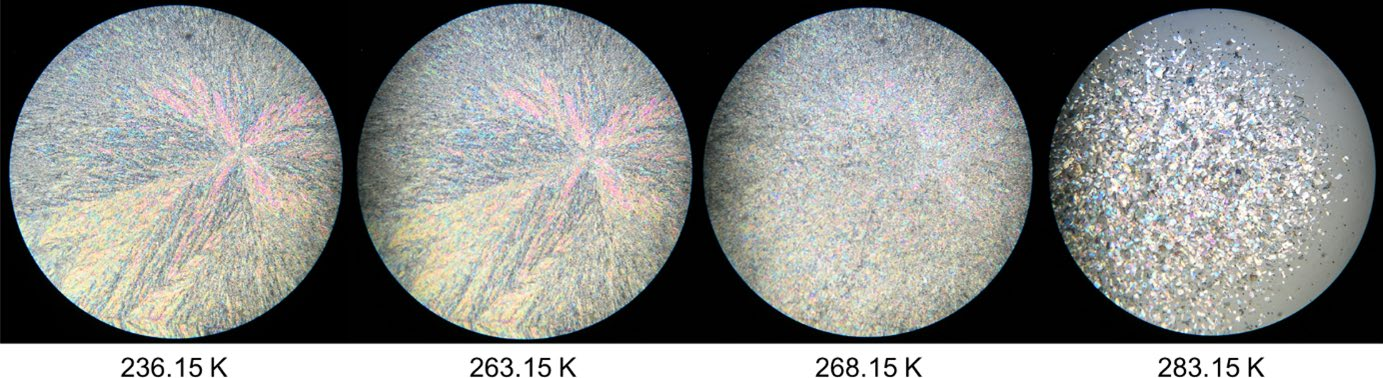
HSM images of dimethyl adipate (DMA) acquired upon heating the sample in the temperature range from 236.15 K to 283.15 K, employing a magnification of 250×

In Fig. 3, the sample appears fully solidified at 236.15 K. At 263.15 K, the first subtle indications of a polymorphic transition begin to appear, manifesting as a faint attenuation in colour. However, it is not until the temperature reaches 268.15 K that the solid phase exhibits a distinctly altered texture and optical character, unmistakably signifying a polymorphic transformation. Finally, at 283.15 K, the sample undergoes a rapid transition to the liquid phase, as shown by the complete disappearance of birefringence in the HSM image.

**Fig. 4:**
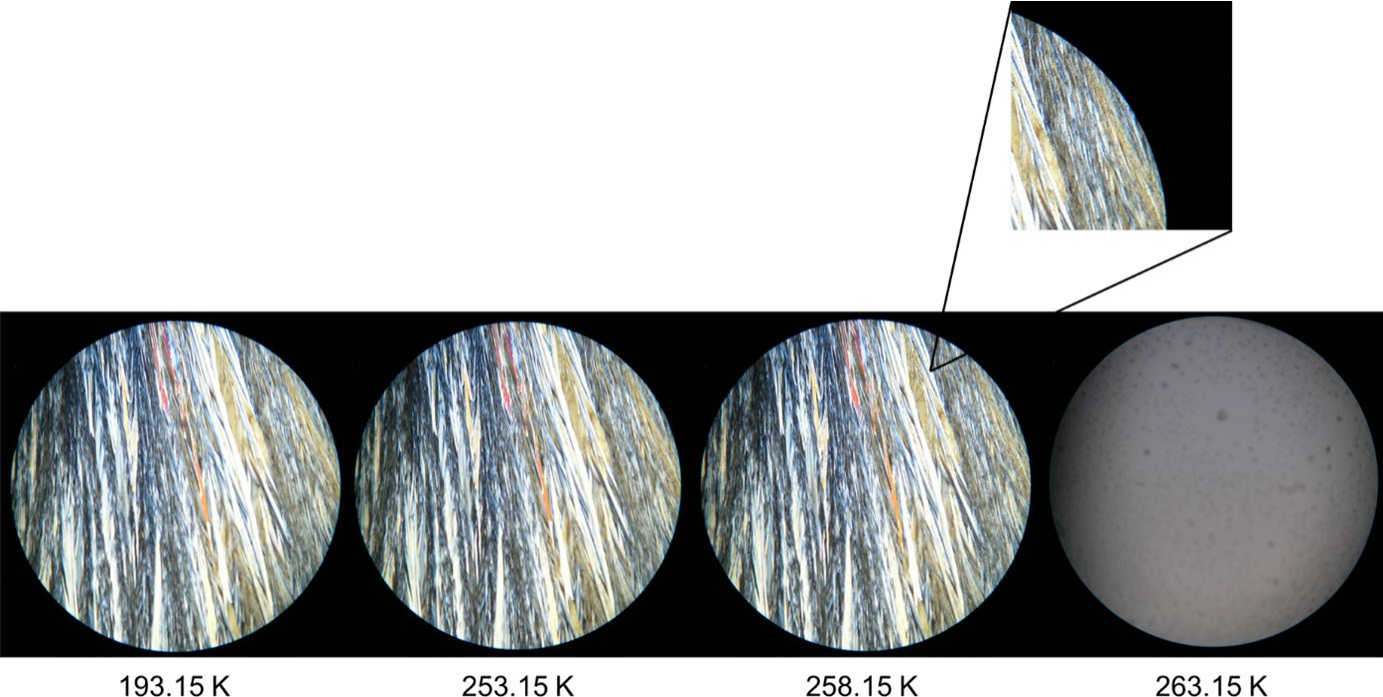
HSM images of dipropyl adipate (DPA) acquired upon heating the sample in the temperature range from 193.15 K to 263.15 K, employing a magnification of 250×

In Fig. 4, the solid-to-liquid phase transition of dipropyl adipate (DPA) is observed between 253.15 K and 263.15 K. The sample is completely solid at 193.15 K, and by 253.15 K, subtle changes suggest the onset of melting, although these are difficult to discern due to image resolution. At 258.15 K, the formation of liquid droplets becomes clearly visible, as highlighted in the zoomed-in image. By 263.15 K, the sample appears fully converted to an isotropic liquid, confirming the completion of the melting process.

**Fig. 5:**
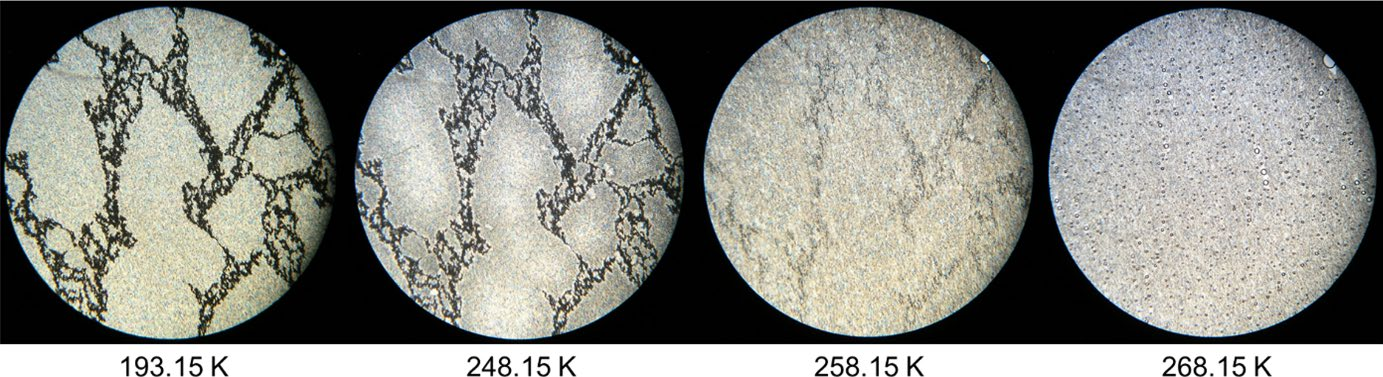
HSM images of the binary mixture with molar fraction x_DPA_ = 0.30 acquired upon heating the sample in the temperature range from 193.15 K to 268.15 K, employing a magnification of 250×

Figure 5 presents the HSM images for the mixture with a molar fraction of x_DPA_ = 0.30, over the temperature range of 193.15 K to 268.15 K. At 193.15 K, the sample is fully solidified, and the first visible changes occur at 248.15 K, indicated by a subtle colour modification suggesting the onset of the solid–liquid phase transition. The transition continues progressively until 268.15 K, as shown in the figure. The phase change is completed at 276.15 K (not shown in the figure).

**Fig. 6:**
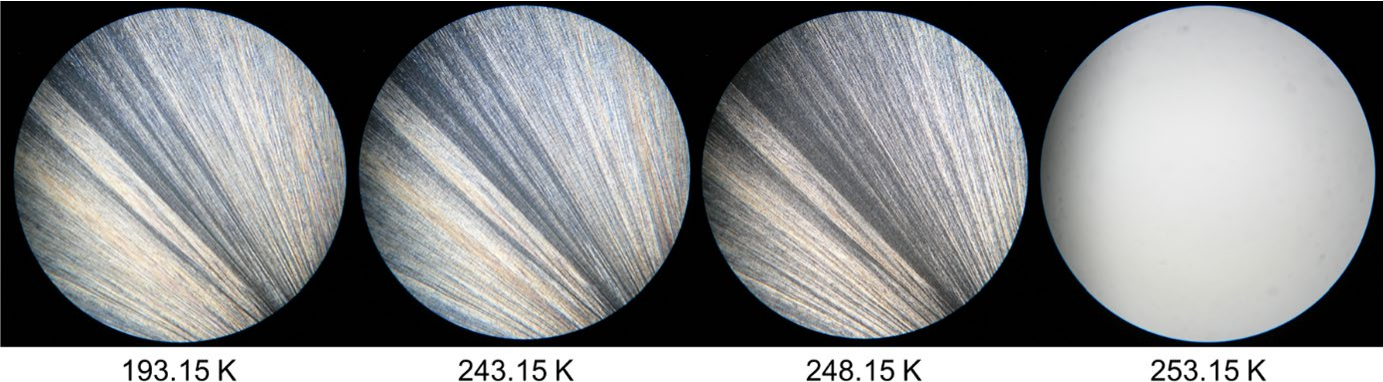
HSM images of the binary mixture with molar fraction x_DPA_ = 0.77 acquired upon heating the sample in the temperature range from 193.15 K to 253.15 K, employing a magnification of 250×

Figure 6 shows the HSM images for the binary mixture with molar fraction x_DPA_ = 0.77, which was identified as the eutectic composition based on the combined results from DSC, HSM, and Raman spectroscopy. The HSM analysis reveals a narrow and well-defined solid–liquid transition, starting at 243.15 K and ending abruptly at 253.15 K, as clearly illustrated in the figure. This sharp transition is characteristic of eutectic behaviour.

**Fig. 7:**
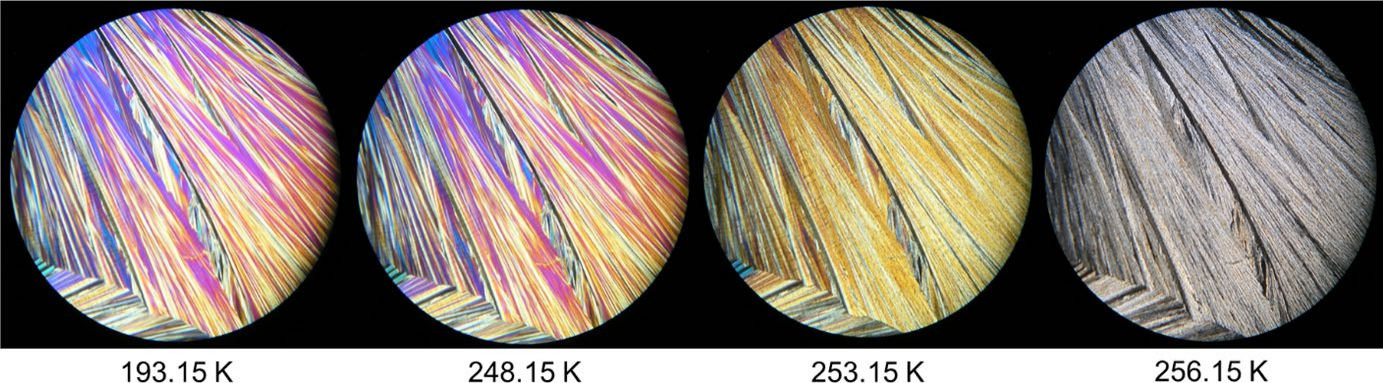
HSM images of the binary mixture with molar fraction x_DPA_ = 0.95 acquired upon heating the sample in the temperature range from 193.15 K to 256.15 K, employing a magnification of 250×

Finally, Fig. 7 displays the HSM images for the binary mixture with molar fraction x_DPA_ = 0.95. At 193.15 K, the sample is fully in the solid state. The first signs of melting appear at 248.15 K, indicated by a subtle colour change, particularly noticeable in the pink region on the right side of the image. By 253.15 K, the sample exhibits a more pronounced colour change and the formation of liquid droplets, visible as small black spots. At 256.15 K, the birefringence disappears, and the liquid phase continues to grow significantly. The sample was observed to be fully liquefied by 257.15 K, although this final image is not included in Fig. 7, as it was not deemed necessary for further analysis.

### Raman Spectroscopy

Raman spectroscopy provides critical information for the construction of binary phase diagrams, particularly in identifying both S–S and S–L phase transitions and characterizing the solid phases themselves.

In the context of phase transitions, this technique is particularly effective in detecting polymorphic transformations and facilitating a reliable assignment of the DSC peaks to S–S or S–L transitions. Additionally, it provides essential information, during an S–L transition, about the nature of the solid phase, enabling the distinction between a solid solution, where the solid phase is a homogeneous mixture of both components (isomorphism), and a mechanical mixture of two distinct solids, each corresponding to one of the pure compounds (non-isomorphism). This distinction is highly valuable for accurately characterizing binary phase diagrams and is particularly relevant for thermal energy storage (TES) applications, where phase separation in the solid state can significantly impact PCM performance.

Given the low-temperature range of the systems investigated in our research, Raman spectroscopy has proven to be the most suitable technique for acquiring the necessary structural information for constructing a phase diagram, as demonstrated in our previous studies [1–4].

**Fig. 8:**
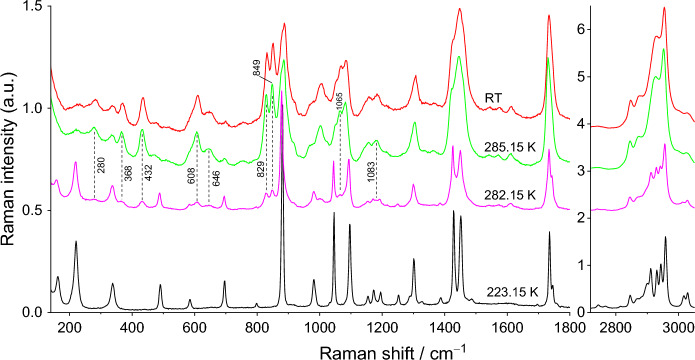
Temperature variation Raman spectra of DMA

**Fig. 9:**
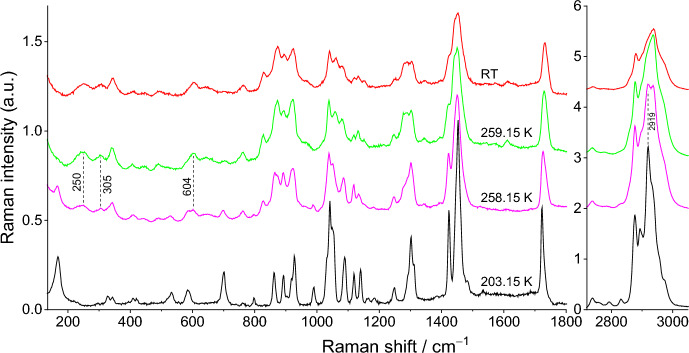
Temperature variation Raman spectra of DPA

**Fig. 10:**
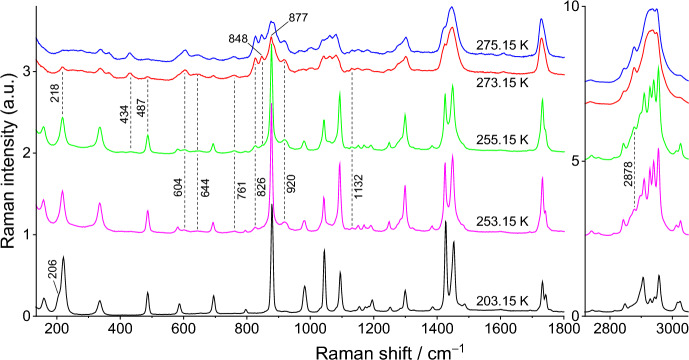
Temperature variation Raman spectra for the mixture x_DPA_ = 0.30

**Fig. 11:**
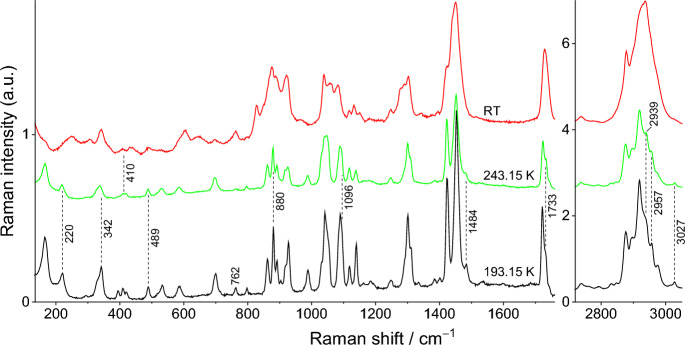
Temperature variation Raman spectra for the mixture x_DPA_ = 0.77

**Fig. 12:**
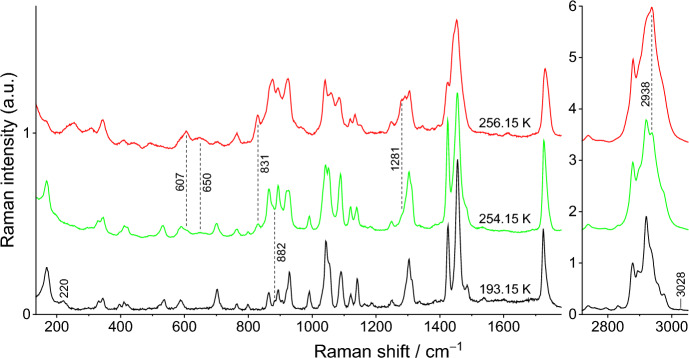
Temperature variation Raman spectra for the mixture x_DPA_ = 0.95

The Raman spectra for pure compounds, DMA and DPA, and for their most relevant binary mixtures (x_DPA_ = 0.30, 0.77, and 0.95) are presented in Figs. 8, 9, 10, 11, and 12. Spectra for the remaining binary compositions studied are available in section S3 of the Supplementary Information. The marker bands used for the identification of each pure compound are given below:

**Dimethyl Adipate (DMA)**

- Solid: 69, 113, 162, 221, 337, 489, 695, 880, 1045, 1095, 1155, 1173, 1194, 1428, 1451, 1735, 1745, 2744, 2766, 2815, 2845, 2867, 2899, 2911, 2930 + 2943, 2958, 3016, and 3028 cm^–1^.
- Liquid: 280, 368, 434, 609, 646, 830, 849, 884, 1002, 1066, 1084, 1157, 1183, 1305, 1446, 1733, 2847, 2874, 2929, 2953, and 3027 cm^–1^.

Raman spectroscopy did not show clear signs of polymorphism, a result that is consistent with DSC results, where only a very subtle event is detected before the solid–liquid phase transition, and with HSM data. The sample is in the solid phase at 223.15 and 280.15 K. The onset temperature of the S–L transformation is 280.15 K and 282.15 K. At 282.15 K, Raman bands related to the liquid form appear at 280, 368, 432, 608, 646, 829, 849, 1065, and 1083 cm^–1^, as well as overall broadening of the spectral feature at 2800–3050 cm^–1^ making it appear more similar to that of the liquid phase. Nevertheless, at this temperature all strong Raman features of the solid phase are still clearly observable. On the other hand, by 285.15 K, all bands of the solid state have disappeared, indicating that the S–L transformation is complete.

**Dipropyl Adipate (DPA)**

- Solid: 74, 168, 532, 585, 701, 798, 862, 892, 918, 928, 989, 1032 (shoulder), 1041, 1050 (shoulder), 1089, 1119, 1140, 1248, 1292 (shoulder), 1302, 1311 (shoulder), 1424, 1453, 1723, 2738, 2792, 2831, 2876, 2893, 2920, 2934 (shoulder), and 2973 (shoulder) cm^–1^.
- Liquid: 250, 305, 344, 605, 764, 828, 874, 895, 923, 1041, 1061, 1082, 1132(triplet), 1294, 1424(shoulder), 1452, 1731, 2739, 2881, and 2937 (wide) cm^–1^.

The first noteworthy indications of the beginning of the S–L transition in the spectra of pure DPA appear at 258.15 K, when the bands of the liquid phase become visible at 250, 305, and 604 cm^–1^ and the peak at 2919 cm^–1^ of the solid phase becomes notably smaller. The phase transition continues to evolve until 259.15 K, when the spectrum becomes equivalent to the room-temperature spectrum, indicating that the sample is fully liquid.

After the identification of the marker bands for each phase of the pure compounds, it became possible to analyse the spectra of their binary mixtures.

For the binary mixture with molar fraction x_DPA_ = 0.30, the spectrum recorded between 203.15 and 252.15 K closely resembles that of pure DMA, exhibiting some minor band shifts and a new shoulder at 206 cm^–1^. Interestingly, despite the relatively high concentration of DPA in the mixture, the contribution of the DPA component to the spectrum remains very minimal. At 253.15 K, the first peaks of liquid DPA appear at 604, 644, 761, 826, 920, 1132, and 2878 cm^–1^. Subsequently, at 255.15 K, very subtle characteristic bands at 434 and 848 cm^–1^, attributed to the DMA liquid phase appear, whereas all peaks of the solid DMA phase persist. As heating continues, the liquid phase bands of the liquid phase gradually grow, while the solid DMA phase bands diminish. At 273.15 K, the last bands of solid DMA are still observed at 218, 487, and 877 cm^–1^, whereas no bands related to solid DPA are discernible. Finally, at 275.15 K, the spectrum of the mixture is equivalent to that obtained at room temperature, indicating the completion of the S–L phase transition.

Regarding the binary mixture with x_DPA_ = 0.77, the spectrum recorded at 193.15 K is mostly dominated by the typical features of solid DPA, with some of the strongest DMA marker bands visible at 220, 342, 489, 880, 1096 (shoulder), 1733 (shoulder), 2957, and 3027 cm^–1^. The first spectral changes are detected already at 243.15 K. Specifically, the triplet of bands at 394–421 cm^–1^ starts transforming into a single broadband centred at 410 cm^–1^, characteristic of the DPA liquid phase. Similar behaviour is observed for the DPA solid bands at 762 and 1484 cm^–1^. Additionally, the marker band of DPA liquid phase appears at 2939 cm^–1^. At 250.15 K, the spectrum is already equivalent to the one obtained at room temperature, which is very similar to that of the pure DPA liquid phase. These results indicate that the S–L phase transition is very fast, and both compounds melt simultaneously as expected for the eutectic composition.

Finally, for the mixture with a composition of x_DPA_ = 0.95, the spectrum taken at 193.15 K is essentially identical to that of solid DPA, with some very minor contribution from the DMA solid phase, evidenced by the very subtle bands at 220, 882 (shoulder), 1775 (shoulder), and 3028 cm^–1^. At 254.15 K, the first modifications related to the beginning of the S–L phase transition become visible in the spectrum. Specifically, bands at 1281 (sh.), and 2938 cm^–1^, characteristic of liquid DPA appear as well as the bands at 609 (shoulder), 650 (broad), and 831 cm^–1^ related to both liquid phases. At 255.15 K, the S–L transition continues to progress, and by 256.15 K, the spectrum is already equivalent to that obtained at room temperature, indicating complete phase transition.

Globally, the Raman spectroscopy results corroborate the DSC findings, demonstrating excellent agreement in temperature values, despite the differences in temperature control between the two techniques. Furthermore, the overall analysis of the studied binary mixtures reveals a non-isomorphic behaviour of the system. This is evidenced by the fact that the marker bands of each compound in the liquid phase appear at different temperatures, indicating the presence of two distinct pure solid phases (DMA and DPA) rather than a solid solution composed of both. Therefore, Raman spectroscopy has proven once again to be essential not only in validating the DSC temperature data but, more importantly, in characterizing this specific feature impacting on the thermal behaviour of the system, an essential step in assessing its potential as a PCM.

## Solid–Liquid Phase Diagram

**Fig. 13:**
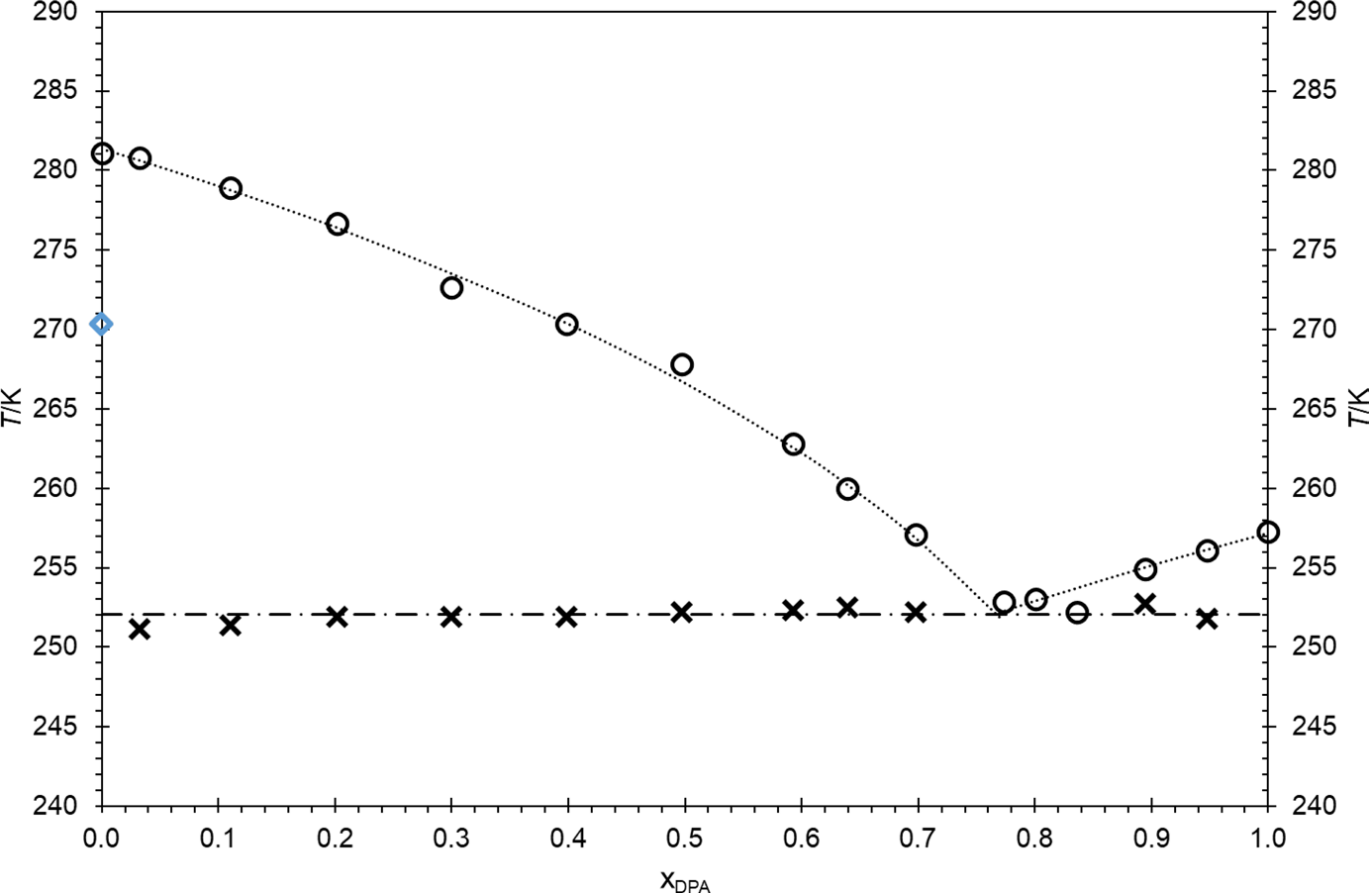
Proposed binary solid–liquid phase diagram for the DMA and DPA mixture. **·····** fitted *liquidus* lines, **– · – · – · –** *solidus* line, **O** experimental *liquidus* data points; ×experimental *solidus* data points, **◊** polymorphic transition for pure DMA

The construction of the binary phase diagram, shown in Fig. 13, is based on the results of the three experimental techniques presented above. Each one of these three techniques gives us different and complementary information that was deemed essential for the design of the solid–liquid phase diagram. The DSC results enable the accurate determination of the temperatures and enthalpies of transitions, either solid–solid or solid–liquid. The HSM enables to assign the DSC peaks to a specific phase transition by visual confirmation. Finally, Raman spectroscopy helped assign the DSC peak to the phase transitions while providing accurate information about the nature of the solid phase itself.

The studied binary system exhibits a relatively simple binary phase diagram with a eutectic behaviour with a eutectic point for x_DPA_ = 0.77 at 252.83 K. Additionally, the binary mixture indicates to be a non-isomorphous system with the solid phase being formed by two different solids, each one corresponding to one of the pure compounds.

The solid–liquid phase diagram represented in Fig. 13 was constructed from experimental data for fourteen binary mixtures and the two pure compounds, totalizing sixteen experimental points. The temperature values shown in Fig. 13 are the *T*_*max*_ values obtained by DSC and presented in Table S1 of section S1 in the Supplementary Information. In particular, the *solidus* line was obtained from the *T*_*max*_ values of the first DSC peak and the *liquidus* line from the *T*_*max*_ values of the second DSC peak, except for the pure compounds and for the binary mixtures exhibiting only one peak which includes, for example, the eutectic composition.

In our previous papers on *n*-alkyl adipates and even *n*-alkanes [1, 2], which demonstrated eutectic behaviour, the *liquidus* lines on these solid–liquid phase diagrams were successfully fitted using Eq. 1.

1 $$\text{ln}\left(\frac{1}{x}\right)=a+b\cdot\frac{1}{T}$$

This equation is in fact a modification of the simplified equation shown by Denbigh [13] for the freezing point depression (Eq. 2).

2 $$\text{ln}\left(\frac{1}{x}\right)\cong \frac{{L}_{Mi}}{R}\left(\frac{1}{T}-\frac{1}{{T}_{Mi}}\right)$$

The details on the obtainment of this equation and its validity for this work are described elsewhere [1, 2, 13].

In light of the approximations employed in Eq. 1, the fitting values of *L*_Mi_ and *T*_Mi_ in Eq. 2 do not precisely correspond to the actual fusion properties, enthalpy, *Δ*_*fus*_*H*, and temperature, *T*_fus_, for the pure compounds. Nevertheless, based on Eqs. 1 and 2, the parameters *L*_*Mi*_ and *T*_*Mi*_ can be derived from [1, 2]:

3 $$a \cong - \frac{{L_{Mi} }}{R} \cdot \frac{1}{{T_{Mi} }}$$

4 $$b \cong \frac{{L_{Mi} }}{R}$$

Additionally, from the fitting of both sides of the diagram, the fitted eutectic point (temperature and composition) can be determined using the intersection of the obtained equations for both sides of the diagram as described previously [1, 2]. The fitted values of *L*_*Mi*_ and *T*_*Mi*_ are listed in Table 4 and compared with the experimental results. For the eutectic point, the fitted eutectic composition and temperature are also compared to the experimental results.

In the present study, the compositions x_DPA_ = 0.77 and 0.84 were excluded from the fitting of the *liquidus* lines. The former corresponds to the experimental eutectic composition and may be associated with either side of the phase diagram, potentially biasing the fit; thus, it was deliberately omitted to better capture the intrinsic behaviour of the remaining data points. The latter composition, x_DPA_ = 0.84, exhibits only a single DSC peak, which lies within the solidus region, rendering it unsuitable for the determination of the *liquidus* line (Table 3).

**Table 3:** Fitted parameters *a* and *b* of Eq. 1, and the absolute root mean square deviation, *rmsd*, to the experimental liquidus data points for the binary system with DMA and DPA

Left side of the diagram			Right side of the diagram		
*a*	*b* (K)	*rmsd* (K)	*a*	*b* (K)	*rmsd* (K)
–12.5620	3.5341 × 10^3^	0.49	– 13.1491	3.3815 × 10^3^	0.10

**Fig. 14:**
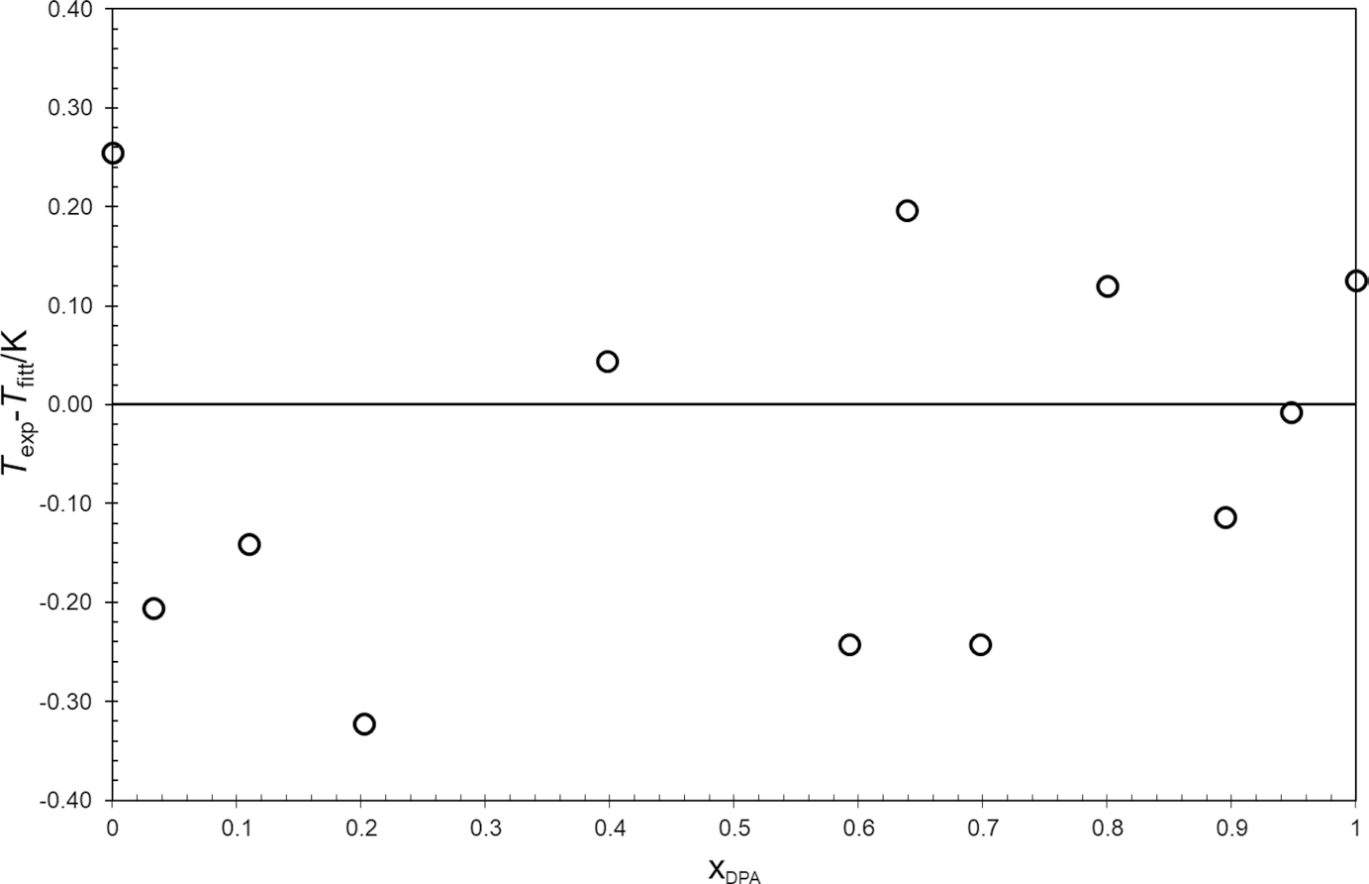
Deviations of the experimental data obtained by thermal analysis (DSC), from the correlation fitting Eq. 1 for the *liquidus* line of the binary system DMA + DPA, as a function of DPA molar fraction, x_DPA_

Nevertheless, the *liquidus* lines on the solid–liquid phase diagram were once again successfully fitted using Eq. 1 and the obtained results show a good agreement with the experimental results for the pure compounds and the eutectic mixture (Table 4), and for the other binary mixtures, considering the deviations represented in Fig. 14. The obtained values for *a* and *b* fitting parameters are presented in Table 3 for both sides of the diagram.

**Table 4:** Comparison of the experimental results of *T*_*fus*_ and *Δ*_*fus*_*H* for pure DMA and DPA with the results for *T*_*Mi*_ and *L*_*Mi*_ obtained by fitting Eq. 1

Dimethyl adipate (DMA)			Dipropyl adipate (DPA)		
*T*_*Mi*_	*T*_*fus*_	Dev. (K)	*T*_*Mi*_	*T*_*fus*_	Dev. (K)
280.02	281.08	– 0.35	261.20	257.29	0.15

L_Mi_ (J·g^−1^)	*Δ*_*fus*_*H* (J·g^−1^)	Dev. (J·g^−1^)	L_Mi_ (J·g^−1^)	*Δ*_*fus*_*H* (J·g^−1^)	Dev. (J·g^−1^)
180.1	168.7	– 11.4	122.8	124.0	1.2

Eutectic point				
x_exp_	*T*_*exp*_ (K)	x_fit_	*T*_*fit*_ (K)	Dev. (K)
0.7733	252.83	0.767	252.08	0.75

**Fig. 15:**
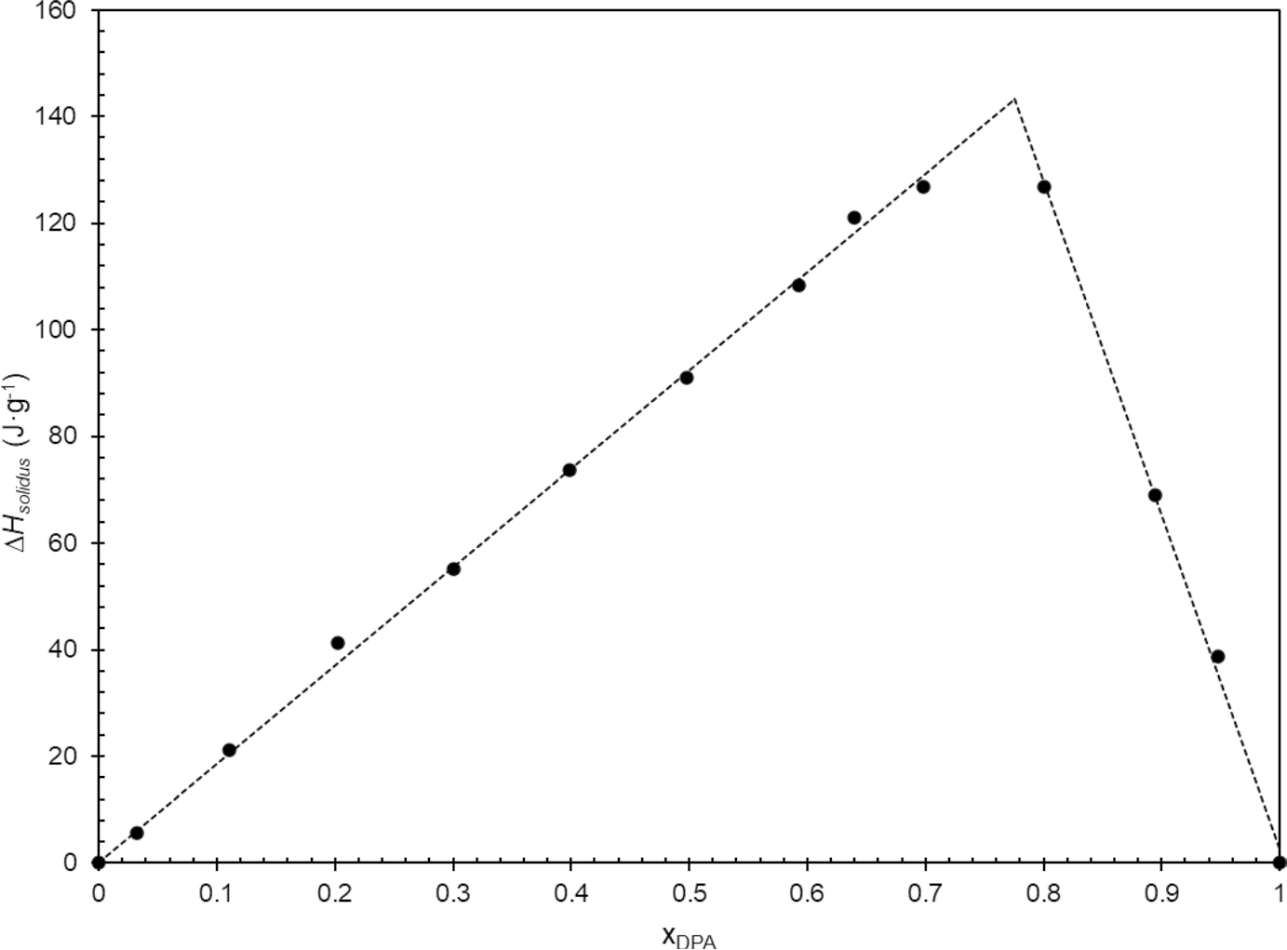
Tammann diagram of the binary system DMA + DPA, as a function of DPA molar fraction, x_DPA_

The Tammann diagram has been widely used and defended by various authors over the years for determining eutectic compositions [14–19]. In fact, the Tammann plot allows for the prediction of enthalpy variations associated with first-order transformations as a function of concentration, based on the lever rule [15]. In practice, the eutectic composition can be directly determined from the Tammann plot [15], which also provides valuable insights into solid solubilities at the eutectic temperature [15, 18, 19]. Figure 15 presents the Tammann diagram, where the experimental enthalpy of the transition at the eutectic temperature is plotted against the molar fraction of dipropyl adipate, x_DPA_.

The dataset used for the construction of the Tammann diagram is detailed in section S5 in the Supplementary Information. From the original experimental dataset only three binary mixtures presented overlapped peaks that required further analysis using the OriginPro® software to perform the peak deconvolution. Also, the binary mixture with molar fraction x_DPA_ = 0.84 is not represented in Fig. 15 and was not used for further calculations because it represented an outlier.

From the Tammann plot it is possible to determine the eutectic composition as x_DPA_ = 0.775 and the corresponding enthalpy of phase transition as 143.3 kJ·mol^−1^. Additionally, this analysis confirms the experimental results regarding the solid phase solubility. Once the straight lines of the Tammann plot intercept *ΔH*_*solidus*_ at x_DPA_ = 0 and x_DPA_ = 1, it can be concluded that there is no miscibility in the solid state between dimethyl adipate and dipropyl adipate. The comparison between experimental results and fitted results (freezing-depression curve and Tammann diagram) for the eutectic point is presented in Table 5.

**Table 5:** Comparison of the experimental results with those obtained from the Tammann diagram and from fitting Eq. 1, for x_eut_, *T*_eut_, and *Δ*_*eut*_*H*

		x_eut_ (DPA)	T_eut_ (K)	*Δ*_*eut*_*H* (J·g^−1^)
Experimental		0.773	252.83	130.8
Freezing-Depression Curve (Eq. 1)		0.786	252.65	–
Tammann		0.775	–	143.3
	|Abs. Deviation Exp|	0.002	–	12.5
	|Abs. Deviation Eq. 1|	0.011	–	–

## Conclusions

Organic compounds are widely recognized for their potential as phase change materials (PCM) due to their high latent heat, chemical stability, and, frequently, also environmental friendliness. Among them, non-paraffin-based compounds such as esters have recently demonstrated promising potential for thermal energy storage (TES) applications, supported by their availability, renewability, and low cost. However, most existing studies focus on moderate to high-temperature ranges, leaving a gap in the development of materials suitable for sub-zero TES applications. To address this gap, one of the objectives of this study was to explore new, effective organic PCM for cold TES technologies.

The present article includes the thermal characterization of the binary system composed of two di-*n*-alkyl adipates, dimethyl (DMA) and dipropyl (DPA) for sub-zero TES applications. DSC tests were performed on sixteen different samples, including the two pure compounds, and complemented with HSM and Raman spectroscopy techniques, allowing the construction of the solid–liquid phase diagram of the binary system. Additionally, the fitting of the *liquidus* line using the freezing-depression curve equation and the Tammann plot are presented. The experimental results reveal a eutectic point characterized by a eutectic composition of x_DPA_ = 0.77 with a eutectic temperature of 252.83 K and an enthalpy of fusion Δ_*fus*_*H* = 130.8 J·g^−1^. The prediction of the eutectic data obtained from the fitting of the *liquidus* curve and by the Tammann plot are in good agreement with the experimental data.

To the best of the authors’ knowledge, the solid–liquid phase diagram of the DMA + DPA system has not been previously reported. Therefore, this study not only offers valuable insights into the potential of this binary mixture for low-temperature TES but also makes a meaningful contribution to the fundamental phase equilibrium research on di-*n*-alkyl adipates by presenting novel experimental data that supports further advancement in the field.

## Supplementary Information

Below is the link to the electronic supplementary material.

Supplementary file1 (DOCX 4039 KB)

## Data Availability

No datasets were generated or analysed during the current study.

## References

[1] M.C.M. Sequeira, B.A. Nogueira, F.J.P. Caetano, H.P. Diogo, J.M.N.A. Fareleira, R. Fausto, Di - alkyl adipates as new phase change material for low temperature energy storage. Int. J. Thermophys. **44**, 165 (2023). 10.1007/s10765-023-03274-3

[2] M.C.M. Sequeira, B.A. Nogueira, F.J.P. Caetano, H.P. Diogo, J.M.N.A. Fareleira, R. Fausto, Solid-liquid phase equilibrium: alkane systems for low-temperature energy storage. Int. J. Thermophys. **45**, 28 (2024). 10.1007/s10765-023-03317-9

[3] M.C.M. Sequeira, T. Nikitin, F.J.P. Caetano, H.P. Diogo, J.M.N.A. Fareleira, R. Fausto, Solid – liquid phase equilibrium of the *n* - nonane + *n* - undecane system for low - temperature thermal energy storage. Int. J. Thermophys. **45**, 117 (2024). 10.1007/s10765-024-03411-6

[4] M.C.M. Sequeira, T. Nikitin, F.J.P. Caetano, H.P. Diogo, J.M.N.A. Fareleira, R. Fausto, Phase equilibrium of *n* - nonane + *n* - decane for low - temperature thermal energy storage: insights into odd – even effects. Int. J. Thermophys. **46**, 60 (2025). 10.1007/s10765-025-03531-7 40060118 10.1007/s10765-025-03531-7 PMC11882680

[5] J.C.F. Diogo, H.M.N.T. Avelino, F.J.P. Caetano, J.M.N.A. Fareleira, Fluid phase equilibria density measurements of compressed dipropyl, dibutyl, bis (2-ethylhexyl) adipates from (293 to 373 K) at pressures up to about 68 MPa. Fluid Phase Equilib. **374**, 9 (2014). 10.1016/j.fluid.2014.04.018

[6] J.C.F. Diogo, H.M.N.T. Avelino, F.J.P. Caetano, J.M.N.A. Fareleira, Viscosity measurements of compressed liquid dipropyl and dibutyl adipates. Fluid Phase Equilib. **395**, 26 (2015). 10.1016/j.fluid.2015.03.016

[7] H.M.N.T. Avelino, C.F. Diogo, F.J.P. Caetano, M.N.A. Fareleira, Viscosity and self-diffusion coefficients of dialkyl adipates: a correlation scheme with predictive capabilities. J. Chem. Eng. Data **60**, 3696 (2015). 10.1021/acs.jced.5b00622

[8] M.J.P. Comuñas, J.P. Bazile, L. Lugo, A. Baylaucq, J. Fernández, C. Boned, Influence of the molecular structure on the volumetric properties and viscosities of dialkyl adipates (dimethyl, diethyl, and diisobutyl adipates). J. Chem. Eng. Data **55**, 9 (2010). 10.1021/je100237h

[9] ŞI. Kirbaşlar, E. Ince, S. Şahin, U. Dramur, (Liquid + liquid) equilibria of (water + propionic acid + dibasic esters) ternary systems. J. Chem. Thermodyn. **39**, 1493 (2007). 10.1016/j.jct.2007.02.012

[10] D. Mondieig, F. Rajabalee, V. Metivaud, H.A.J. Oonk, M.A. Cuevas-Diarte, n-alkane binary molecular alloys. Chem. Mater. **16**, 786 (2004). 10.1021/cm031169p

[11] S.N. Gunasekara, V. Martin, J.N. Chiu, Phase equilibrium in the design of phase change materials for thermal energy storage: state-of-the-art. Renew. Sustain. Energy Rev. **73**, 558 (2017). 10.1016/j.rser.2017.01.108

[12] J.J. Moura Ramos, R. Taveira-Marques, H.P. Diogo, Estimation of the fragility index of indomethacin by DSC using the heating and cooling rate dependency of the glass transition. J. Pharm. Sci. **93**, 1503 (2004). 10.1002/jps.20061 10.1002/jps.20061 15124208

[13] K. Denbigh, *Principles of chemical equilibrium*, 2nd edn. (Cambridge University Press, London, 1966)

[14] J. Guenet, Contributions of phase diagrams to the understanding of organized polymer-solvent systems. Thermochim. Acta, **284**, 67 (1996). 10.1016/0040-6031(96)02892-4

[15] A. Dańczak, L. Rycerz, Reinvestigation of the DyCl3–LiCl binary system phase diagram. J. Therm. Anal. Calorim. **126**, 299 (2016). 10.1007/s10973-016-5547-y

[16] L. Rycerz, Practical remarks concerning phase diagrams determination on the basis of differential scanning calorimetry measurements. J. Therm. Anal. Calorim. **113**, 231 (2013). 10.1007/s10973-013-3097-0

[17] D. Wei, S. Han, X. Shen, Solid – liquid phase equilibria of (n-octadecane with myristic, and palmitic acid) binary mixtures used as phase change materials (PCMs). J. Chem. Thermodyn. **101**, 7 (2016). 10.1016/j.jct.2016.05.008

[18] Y. Corvis, P. Négrier, M. Lazerges, S. Massip, J. Léger, P. Espeau, Lidocaine / L-menthol binary system : cocrystallization versus solid-state immiscibility. J. Phys. Chem. B **114**, 5420 (2018). 10.1021/jp101303j 10.1021/jp101303j 20377180

[19] L.A.D. Boros, M.L.S. Batista, J.A.P. Coutinho, M.A. Krähenbühl, A.J.A. Meirelles, M.C. Costa, Binary mixtures of fatty acid ethyl esters: Solid-liquid equilibrium. Fluid Phase Equilib. **427**, 1 (2016). 10.1016/j.fluid.2016.06.039

