# Supplementary material for: Solid-Liquid Phase Diagram of the Dimethyl + Dipropyl Adipates System: Application to Low-Temperature Thermal Energy Storage
Source: Int J Thermophys. 2025 Jul 10;46(9):131. doi: 10.1007/s10765-025-03598-2 (PMC12241232; doi:10.1007/s10765-025-03598-2)
Supplement: Supplementary file 1 — Supplementary file1 (DOCX 4039 KB) [file 10765_2025_3598_MOESM1_ESM.docx]

**Supplementary Information for**

**Solid–Liquid Phase Diagram of the Dimethyl+Dipropyl Adipates System: Application to Low–Temperature Thermal Energy Storage**

Maria C.M. Sequeira^1^, Timur Nikitin^2^, Fernando J.P. Caetano^3,4*^, Hermínio P. Diogo^1*^, João M.N.A. Fareleira^1^, Rui Fausto^2,5^

^1^ Centro de Química Estrutural, Institute of Molecular Sciences, Departamento de Engenharia Química, Instituto Superior Técnico, Universidade de Lisboa, Av. Rovisco Pais, 1049-001 Lisboa, Portugal

^2^ CQC-IMS, Departamento de Química, Universidade de Coimbra, 3004-535 Coimbra, Portugal

^3^ Departamento de Ciências e Tecnologia, Universidade Aberta, 1269-001 Lisboa, Portugal

^4^ Centro de Química Estrutural, Institute of Molecular Sciences, Instituto Superior Técnico, Universidade de Lisboa, Av. Rovisco Pais, 1049-001 Lisboa, Portugal

^5^ ERA-Chair Spectroscopy@IKU, Faculty of Sciences and Letters, Dept. of Physics, Istanbul Kultur Univ., Ataköy Campus, Bakirköy 34156, Istanbul, Turkey

*Corresponding Author: fernando.caetano@uab.pt; hdiogo@tecnico.ulisboa.pt

**Contents**

**Section S1: DSC data…………………………………………………..............................................3**

- **Fig. S1** DSC heating curves of all studied binary mixtures, with compositions indicated by the *x*_DPA_ molar fraction. The scanning rate was *β* = 5 K·min^–1^ (exo up). Two consecutive heating runs are presented for each composition.
- **Table S1** DSC data for pure dimethyl adipate (DMA), pure dipropyl adipate (DPA) and for all of their studied binary mixtures, including the onset temperatures, *T_onset_*, maximum peak temperatures, *T_max_*, and the corresponding enthalpies of fusion, Δ_fus_*H*, at atmospheric pressure, 0.1 MPa. The scanning rate was *β* = 5 K·min^–1^.

**Section S2: HSM data…………………………….…………………………...……….…………..…...4**

- **Fig. S2** HSM images of the binary mixture with molar fraction x_DPA_=0.60 acquired upon heating the sample in the temperature range from 193.15 K to 269.15 K, employing a magnification of 250×.
- **Fig. S3** HSM images of the binary mixture with molar fraction x_DPA_=0.70 acquired upon heating the sample in the temperature range from 193.15 K to 255.15 K, employing a magnification of 250×.
- **Fig. S4** HSM images of the binary mixture with molar fraction x_DPA_=0.90 acquired upon heating the sample in the temperature range from 193.15 K to 255.15 K, employing a magnification of 250×.

**Section S3: Raman Spectroscopy data………………………………………….…………..……...5**

- **Fig. S5** Temperature variation Raman spectra of the binary mixture with molar fraction x_DPA_=0.50_._
- **Fig. S6** Temperature variation Raman spectra of the binary mixture with molar fraction x_DPA_=0.85_._
- **Fig. S7** Temperature variation Raman spectra of the binary mixture with molar fraction x_DPA_=0.90_._

**Section S4: Statistical Parameters and data……………………………………………..………...8**

**Section S5: Tammann Data………………………………………………………………………..…..8**

- **Fig. S8** Deconvolution of the experimental DSC peak for the binary mixture x_DPA_=0.95 using the software OriginPro®. **▬** experimental DSC results; –– best fitting to the experimental DSC results; –– deconvoluted peaks; –– baseline.
- **Table S2** Extracted data for the ratio of the peak areas, total enthalpy of the peak from DSC experiments, Δ*H*_T_, and the enthalpy of the eutectic transition, Δ*H*_solidus_, from OriginPro® for the three binary mixtures x_DPA_=0.64, 0.90 and 0.95

**S1. DSC Data**

**
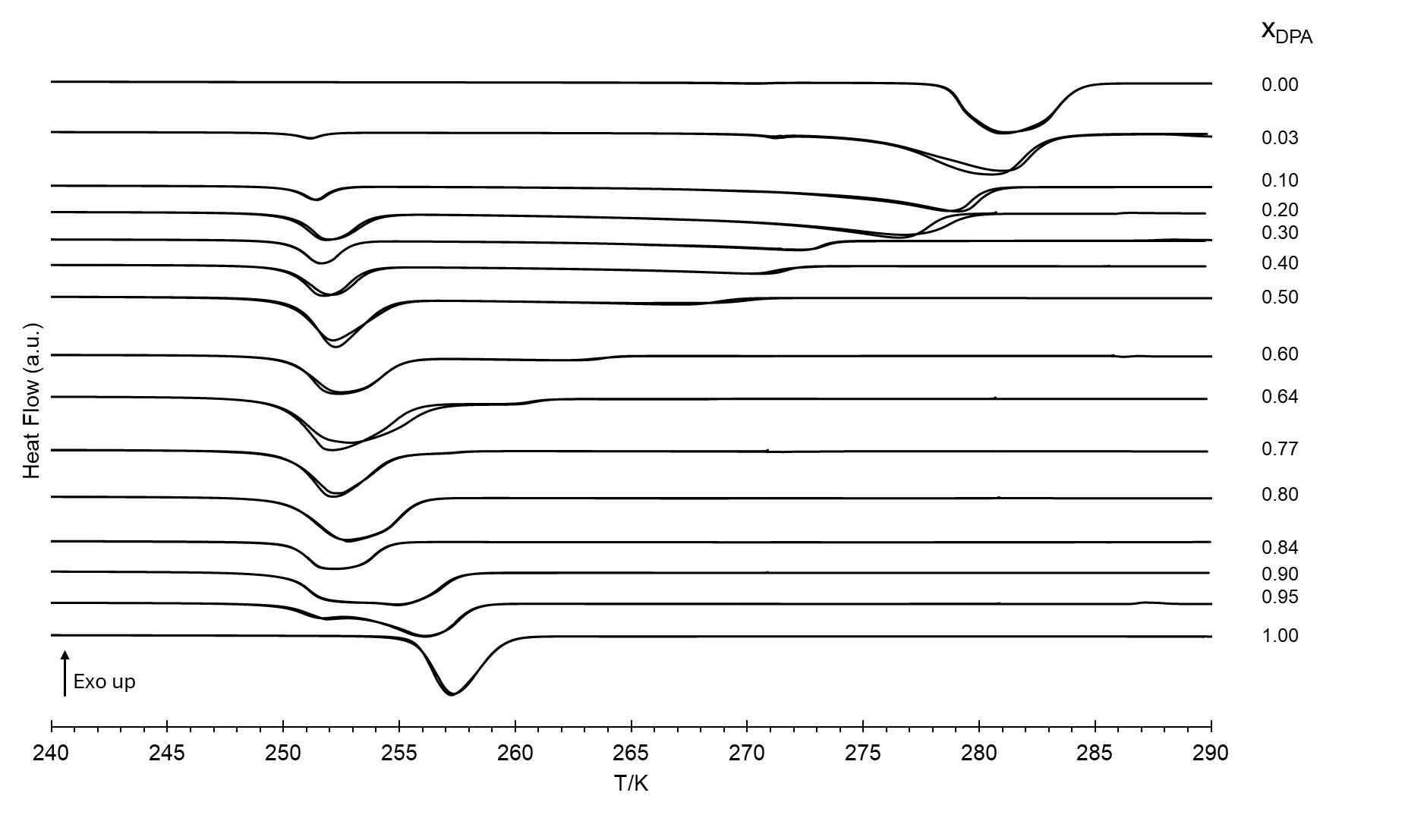
Fig. S1** DSC heating curves of all studied binary mixtures, with compositions indicated by the *x*_DPA_ molar fraction. The heat scan rate was *β* = 5 K·min^–1^ (exo up). Two consecutive heating runs are presented for each composition.

**Table S1** DSC data for pure dimethyl adipate (DMA), pure dipropyl adipate (DPA) and for all of their studied binary mixtures, including the onset temperatures, *T_onset_*, maximum peak temperatures, *T_max_*, and the corresponding enthalpies of fusion, Δ_fus_*H*, at atmospheric pressure, 0.1 MPa. The heating scan rate was *β* = 5 K·min^–1^.

| ***X*_DPA_** | **DSC 1^st^ peak** | | | | | **DSC 2^nd^ peak** | | |
| --- | --- | --- | --- | --- | --- | --- | --- | --- |
|  | ***T*_onset_ /K** | ***T*_max_ /K** | | | **Δ_fus_*H* /J·g^–1^** | ***T*_onset_ /K** | ***T*_max_ /K** | **Δ_fus_*H* /J·g^–1^** |
| 0 | 268.49 | | 270.35 | 0.9 | | 278.62 | 281.08 | 167.9 |
| 0.0326 | 250.03 | | 251.16 | 5.6 | | 275.46 | 280.8 | 126.1 |
| 0.1100 | 250.19 | | 251.40 | 21.2 | | 273.29 | 278.89 | 136.9 |
| 0.2021 | 250.28 | | 251.88 | 41.3 | | 269.10 | 276.69 | 97.9 |
| 0.3000 | 250.14 | | 251.91 | 55.2 | | 265.67 | 272.65 | 64.2 |
| 0.3982 | 250.16 | | 251.88 | 73.7 | | 262.31 | 270.36 | 46.9 |
| 0.4974 | 250.39 | | 252.18 | 90.9 | | 258.68 | 267.81 | 22.8 |
| 0.5930 | 250.26 | | 252.33 | 108.4 | | 257.80 | 262.79 | 8.8 |
| 0.6396 | 249.81 | | 252.49 | 139.2 | | – | – | – |
| 0.6981 | 250.17 | | 252.21 | – | | – | 257.09 | 126.9 ^a)^ |
| 0.7733 | 250.22 | | 252.83 | 130.8 | | – | – | – |
| 0.8003 | 250.21 | | 253.00 | 126.8 | | – | – | – |
| 0.8357^b)^ | 250.33 | | 252.19 | 127.3 | | – | – | – |
| 0.8946 | 250.16 | | 252.76 | – | | – | 254.89 | 130.2 ^a)^ |
| 0.9477 | 249.91 | | 251.8 | – | | 252.2 | 256.11 | 129.3 ^a)^ |
| 1 | 255.71 | | 257.29 | 124.0 | | – | – | – |

^a)^ Enthalpy value for the overlapped peaks; ^b)^ The DSC thermogram indicates that this composition corresponds to the *liquidus* line as attributed in the solid-liquid phase diagram (Fig. 13); Expanded uncertainties for a 95% confidence level (*k* = 2): *U*(*x*) = 0.00016; *U*(*T*) = 0.16 K; *U*(Δ_fus_*H)* = 3.4 J·g^–1^ (see section S4).


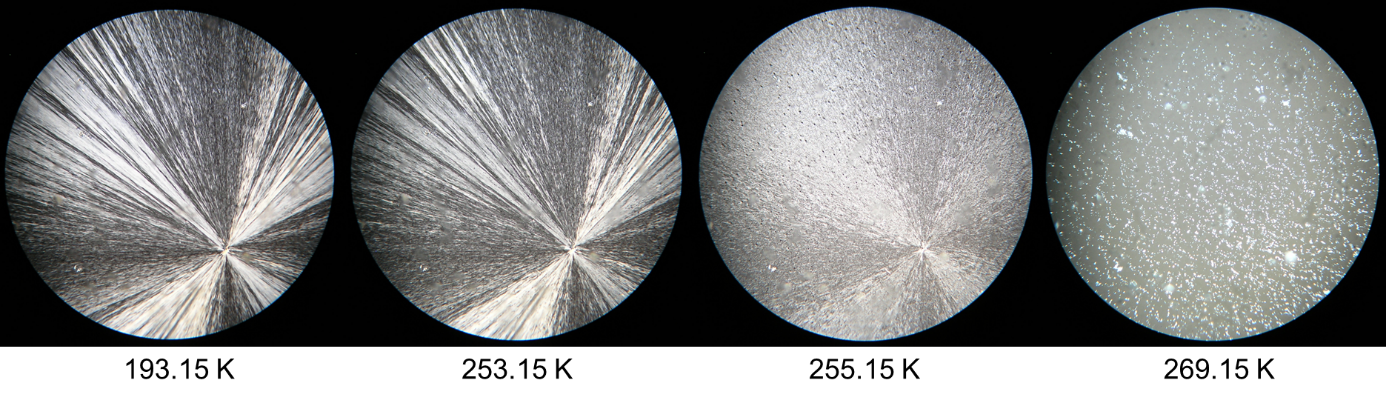
**S2. HSM Data**

**Fig. S2** HSM images of the binary mixture with molar fraction x_DPA_=0.60 acquired upon heating the sample in the temperature range from 193.15 K to 269.15 K, employing a magnification of 250×.


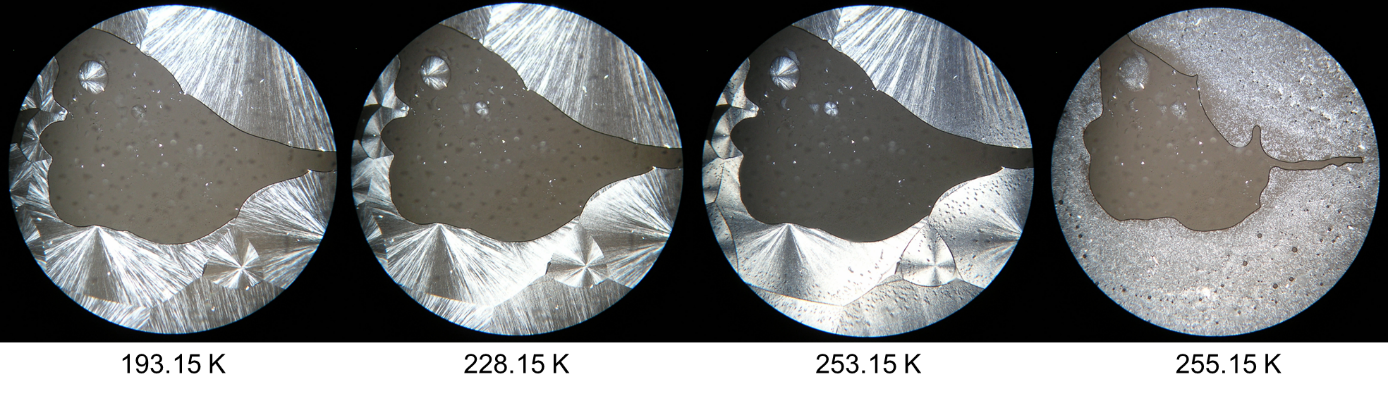


**Fig. S3** HSM images of the binary mixture with molar fraction x_DPA_=0.70 acquired upon heating the sample in the temperature range from 193.15 K to 255.15 K, employing a magnification of 250×.


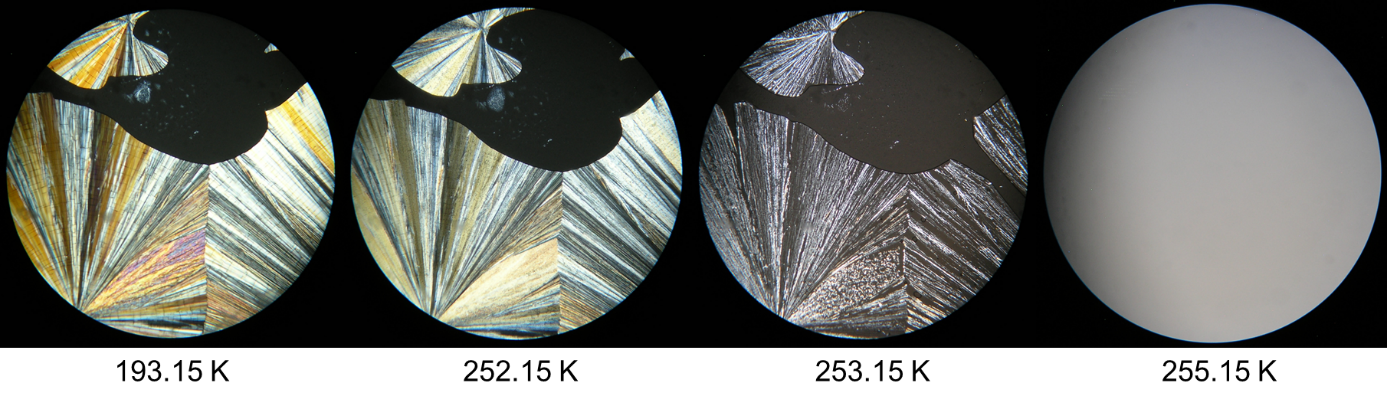


**Fig. S4** HSM images of the binary mixture with molar fraction x_DPA_=0.90 acquired upon heating the sample in the temperature range from 193.15 K to 255.15 K, employing a magnification of 250×.

**S3. Raman Spectroscopy**

In this section the remaining Raman spectroscopy results not shown in the main text of this article are presented. Particularly, the results for the binary mixtures with compositions x_DPA_= 0.50, 0.85 and 0.90 are presented in Figs. S5 – S7.

For the binary mixture x_DPA_= 0.50, at 203.15 K, the spectrum of the mixture is a superposition of those of DMA and DPA components. The first small changes in the spectra are observed at 252.15 K. Specifically, the band at 828 cm^–1^ appears, which is most probably related to the liquid phase of DPA. Indeed, the fusion temperature of DPA is much lower compared to that of DMA. Also, the bands at 926 and 1722 cm^–1^, related to DPA solid phase, decrease. At 253.15 K, more drastic changes are observed. Specifically, the disappearance or drastic decrease of the triplet at 394–420 cm^–1^, bands at 532, 862, 926, 1132 (shoulder), 1138, 1401, 1722, 2898, 2917, and 2975 cm^–1^, all ascribable to the DPA solid phase. Concurrently, broad bands of the liquid state of DPA appear at 605, 762, 1061, and 1284 (shoulder) cm^-1^. At 255.15 K, the first marker bands of the liquid phase of DMA emerge at 433 and 850 cm^–1^. At 263.15 K, the spectrum is practically equivalent to that obtained at room temperature, but the melting of DMA has not yet finished. Indeed, some Raman bands related to the DMA liquid phase are missing: the shoulder at 887 cm^–1^ and the band at 1004 cm^–1^, while a band of solid DMA is still discernible at 488 cm^–1^, with a very small intensity. Nevertheless, the spectrum at this temperature is practically equivalent to that at room temperature. At 267.15 K, the spectrum becomes equivalent to that of the liquid phase.

**Fig. S5** Temperature variation Raman spectra of the binary mixture with molar fraction x_DPA_=0.50

The mixture x_DPA_= 0.85 shows that below 251.15 K, similarly to the mixture x_DPA_= 0.80, the spectrum is mostly dominated by the Raman bands of solid DPA, with some of the strongest marker bands of DMA also visible at 220, 343, 489, 881, 1735 (shoulder), 2957 (shoulder), and 3027 cm^–1^. At 251.15 K, it is already possible to observe some changes in the spectrum, although very subtle, with the bands at 220 and 2957 cm^–1^, related to solid DMA, decreasing of intensity. Also, the following bands of solid DPA decrease/broaden: 395, 1484, and 2895 cm^–1^. At 252.15 K, the spectrum of the mixture is already very similar to that of the liquid phase of DPA but still exhibits characteristic marker bands for the solid phase of both compounds. Due to the high concentration of DPA in the mixture, it is possible to observe mostly marker bands characteristic of the liquid phase of DPA at 250, 305, 408, 604, 648, 763, 923, 965, 1040, 1280, and 2937 cm^–1^, together with a very small band of DMA at 849 cm^–1^. At 254.15 K, the spectrum is already equivalent to that obtained at room temperature. As in the previous binary mixture, the S–L transition is very fast, and both compounds melt at the same time. Although it is not the eutectic composition, the behavior is very similar.

**Fig. S6** Temperature variation Raman spectra of the binary mixture with molar fraction x_DPA_=0.85

Finally, for the binary sample with x_DPA_= 0.90, below 252.15 K, the spectrum of the mixture is dominated by Raman bands of DPA, due to its high concentration. However, some strong features of DMA are also observed at 217, 342, 489, and 880 cm^–1^. At 252.15 K, the first signs of phase transition occur with the decrease of the marker bands of the DPA solid phase at 533, 988, 2893 and 2973 cm^–1^ and with the appearance of the marker bands for the DPA liquid phase at 250, 602, growth of 828, 1278 (shoulder) cm^–1^. At 255.15 K, the spectrum is very similar to that of the liquid phase of DPA, with some residual features of the solid phase still visible at 165 (DPA, DMA), 697 (DPA, DMA), 863 (DPA, DMA), 988 (DPA), 1085 (DPA), and 1421 (DPA) cm^–1^. At 256.15 K, the spectrum is equivalent to the one obtained at room temperature.

**Fig. S7** Temperature variation Raman spectra of the binary mixture with molar fraction x_DPA_=0.90

**S4. Statistical Data**

The statistical parameter *σ* (standard deviation) was used to calculate the expanded uncertainty of the temperatures and enthalpies of fusion in this work, as done before in our previous studies [1,2,3], and is defined by Eq. S1,

(S1)

$$\sigma=\left[ \frac{{\sum(X_{i}-\bar{X})}^{2}}{N-1} \right]^{1/2}$$

where *N* is the total number of experimental data points, the subscript *i* stands for the *i-th* experimental point, and *X* stands for temperature or enthalpy values.

The expanded uncertainty *U* is calculated according to Eq. S2:

(S2)

$$U=2\sigma$$

The estimation of the expanded uncertainties for temperatures (*T_onset_* and *T_max_*) and the enthalpy of fusion (Δ_fus_*H*) is based on five independent measurements using the experimental procedure described in section 2.2.1. The five prepared samples correspond to the binary mixture with composition *x*_DPA_= 0.80, since it only presents one very well–defined DSC peak. This estimation is assumed to be valid for all the experimental measurements.

The estimation of the expanded uncertainties for molar fractions (*x*_DMA_ and *x*_DPA_) is based on the error propagation analysis, considering the weighing of the samples and the uncertainty of the molecular weights of these compounds. For the estimation of the uncertainty of molecular weights, the NIST Technical Note 1900 [4] and the IUPAC Technical Report published in 2022 were used [5].

**S5. Tammann Data**

The Tammann diagram was constructed using the experimental data obtained via differential scanning calorimetry (DSC). However, in the case of three of the binary mixtures under investigation, overlapping thermal events were observed, requiring further analytical treatment to enable the accurate determination of the eutectic transition enthalpy, which is a requisite parameter for the development of the Tammann plot.

The experimental results for the binary mixtures with composition x_DPA_= 0.64, 0.90 and 0.95 were analysed using the software OriginPro® (Table S2), and the DSC peaks were deconvoluted, as illustrated in Fig. S8 for the binary mixture x_DPA_= 0.95.


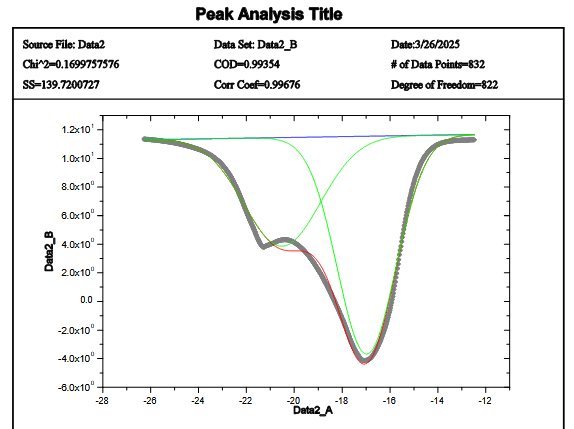


**Fig. S8** Deconvolution of the experimental DSC peak for the binary mixture x_DPA_=0.95 using the software OriginPro®. **▬** experimental DSC results; –– best fitting to the experimental DSC results; –– deconvoluted peaks; –– baseline.

**Table S2** Extracted data for the ratio of the peak areas, total enthalpy of the peak from DSC experiments, Δ*H*_T_, and the enthalpy of the eutectic transition, Δ*H*_solidus_, from OriginPro® for the three binary mixtures x_DPA_=0.64, 0.90 and 0.95.

|  | **x_DPA_=0.64** | **x_DPA_=0.90** | **x_DPA_=0.95** |
| --- | --- | --- | --- |
| **Ratio of the areas** | 87/13 | 53/47 | 30/70 |
| **Total Enthalpy, Δ*H*_T_** | 139.2 | 130.2 | 129.3 |
| **Enthalpy at eutectic transition, Δ*H*_solidus_** | 121.1 | 69.0 | 38.8 |

**References**

[1] M. C. M. Sequeira, B. A. Nogueira, F. J. P. Caetano, H. P. Diogo, J. M. N. A. Fareleira, R. Fausto, Solid–Liquid Phase Equilibrium: Alkane Systems for Low-Temperature Energy Storage, *Int J Thermophys* **45**, 28 (2024). https://doi.org/10.1007/s10765-023-03317-9.

[2] M. C. M. Sequeira, T. Nikitin, F. J. P. Caetano, H. P. Diogo, J. M. N. A. Fareleira, R. Fausto, Solid – Liquid Phase Equilibrium of the *n* ‑ Nonane + *n* ‑ Undecane System for Low ‑ Temperature Thermal Energy Storage. *Int J Thermophys* **45**, 117 (2024). https://doi.org/10.1007/s10765-024-03411-6.

[3] M. C. M. Sequeira, T. Nikitin, F. J. P. Caetano, H. P. Diogo, J. M. N. A. Fareleira, R. Fausto, Phase Equilibrium of *n*-Nonane + *n*-Decane for Low-Temperature Thermal Energy Storage: Insights into Odd–Even Effects. *Int J Thermophys* **46**, 60 (2025).

[4] A. Possolo, Simple Guide for Evaluating and Expressing the Uncertainty of NIST Measurement Results NIST Technical Note 1900, *Natl Inst Stand Technol* **1297**, 1–20 (2015). http://dx.doi.org/10.6028/NIST.TN.1900.

[5] T. Prohaska, J. Irrgeher , J. Benefield , J. K. Böhlke , L. A. Chesson , T. B. Coplen , T. Ding, P. J. H. Dunn, M. Gröning, N. E. Holden, H. A. J. Meijer, H. Moossen, A. Possolo, Y. Takahashi, J. Vogl, T. Walczyk, J. Wang, M. E. Wieser, S. Yoneda, X. Zhu, Juris Meija, Standard atomic weights of the elements 2021 (IUPAC Technical Report). *Pure Appl Chem* **94**, 573–600 (2022). https:// doi.org/ 10.1515/pac-2019-0603.
